# A cross‐sectional review of policies on conflicts of interest and funding in the development manuals of practice guidelines

**DOI:** 10.1002/gin2.70020

**Published:** 2025-04-10

**Authors:** Yangqin Xun, Qiangqiang Guo, Zijun Wang, Akihiko Ozaki, Ying Zhu, Nan Yang, Yajia Sun, Shouyuan Wu, Mengjuan Ren, Ping Wang, Hui Liu, Hui Lan, Yunlan Liu, Qianling Shi, Susan L. Norris, Ivan D. Florez, Joseph L. Mathew, Myeong Soo Lee, Yaolong Chen, Janne Estill

**Affiliations:** ^1^ School of Population Medicine and Public Health Chinese Academy of Medical Sciences & Peking Union Medical College Beijing China; ^2^ Evidence‐Based Medicine Center, School of Basic Medical Sciences Lanzhou University Lanzhou China; ^3^ Institute of Global Health University of Geneva Geneva Switzerland; ^4^ School of Public Health Lanzhou University Lanzhou China; ^5^ Breast and Thyroid Center Jyoban Hospital of Tokiwa Foundation Iwaki City Japan; ^6^ Health Research Methods, Evidence, and Impact (HEI) McMaster University Hamilton Ontario Canada; ^7^ The First School of Clinical Medicine Lanzhou University Lanzhou China; ^8^ Oregon Health & Science University Portland Oregon USA; ^9^ Department of Pediatrics University of Antioquia Medellin Colombia; ^10^ School of Rehabilitation Science McMaster University Hamilton Ontario Canada; ^11^ Pediatric Intensive Care Unit Clínica Las Americas‐AUNA Medellin Colombia; ^12^ Advanced Pediatrics Centre Postgraduate Institute of Medical Education and Research (PGIMER) Chandigarh India; ^13^ Clinical Research Division Korea Institute of Oriental Medicine Daejeon Korea; ^14^ Korean Convergence Medicine University of Science and Technology Daejeon Korea; ^15^ Institute of Health Data Science Lanzhou University Lanzhou China; ^16^ School of Basic Medical Sciences, Research Unit of Evidence‐Based Evaluation and Guidelines, Chinese Academy of Medical Sciences (2021RU017) Lanzhou University Lanzhou China; ^17^ WHO Collaborating Centre for Guideline Implementation and Knowledge Translation Lanzhou China

**Keywords:** conflicts of interest, funding, practice guidelines

## Abstract

**Importance:**

Policies on conflicts of interest (COI) and funding are essential to reduce the risk of bias in the guideline development process.

**Objective:**

To collate and review the content related to COI and funding policies from guideline development handbooks.

**Study design and setting:**

We searched PubMed from its inception until September 10, 2021, websites of key guideline development organizations and Google for guideline development manuals that included COI or funding policies, and performed a cross‐sectional review.

**Results:**

Fifty‐seven guideline development manuals were included. Amongst the 54 handbooks containing a COI policy, all required disclosure of interests. Nineteen (35.2%) manuals defined what constitutes a COI, and 52 (96.3%) specified who should disclose their interests. Thirty‐four (63.0%) manuals recommended an assessment of disclosed interests to determine whether a COI existed, and all of these specified who should perform this review. Thirty‐five (64.8%) manuals addressed the management of COI, of which 26 (74.3%) indicated who should manage COI and 29 (82.9%) reported specific management measures. Twenty‐eight (51.8%) manuals addressed the publication of COI, all recommending that these be publicly accessible. Of the 28 manuals that provided guidance on funding, eight (28.6%) required reporting of funding sources; 14 (50.0%) required that the guideline authors state that the funders' perspectives and interests did not affect the final recommendations; eight (28.6%) specified which kind of funding the guidelines should not accept; and five (17.9%) recommended that the role of funders be restricted.

**Conclusions:**

Policies in guideline manuals report a variety of different elements related to COI and funding. However, a considerable part of the policies did not report precisely what constitutes a COI, the key steps for COI management, or address the sources, influence and acceptability of funding.

## Key points

**Question:** How do conflicts of interest (COI) and funding policies differ across guideline development manuals?

**Findings:** This cross‐sectional review of COI and funding policies from 54 guideline development organizations' development manuals found that COI policies mainly cover four aspects: disclosures of interest, assessment of disclosure of interests, management of COI, and publication of COI. Funding policies addressed primarily the declaration, influence, and restrictions related to funding. However, there were obvious differences between the COI and funding policies in different manuals.

**Meaning:** These findings suggest that discrepancies in COI and funding related policies may lead to lack of transparency, and the situation needs to be improved.

## 1 INTRODUCTION

Clinical practice guidelines (CPG) are statements that include recommendations intended to optimize patient care, informed by a systematic review of evidence and an assessment of the benefits and harms of alternative care options by discussion and consensus.^1^, ^2^, ^3^ High‐quality CPGs can standardize medical workers' actions on providing diagnoses and treatment, narrow the practice gap, improve the quality of medical care, and reduce costs.^4^, ^5^, ^6^, ^7^, ^8^

Any factor that may affect or reasonably be perceived to affect a guideline contributor's objectivity, fairness and independence of financial, personal or professional interests in the process of CPG development could constitute a conflict of interest (COI). Conflicts of interest are a major source of potential bias in guidelines development: they can potentially affect any step in the guideline development process, including the constitution of the guideline development group, the definition of the scope and the key questions, the retrieval and evaluation of evidence, the formulation of recommendations, and the dissemination, implementation and assessment of the guideline.^1^, ^9^, ^10^ They may subsequently lead to an overestimation of beneficial effects or an underestimation of harms, thus seriously affecting the validity or trustworthiness of the recommendations.^2^, ^3^, ^11^, ^12^ Problems related to COI have been demonstrated even among the most well‐recognized and authoritative professional guideline development institutions.^13^, ^14^, ^15^ COI has been shown to influence development of guidelines for oncology, acute ischemic stroke, endocrinology, and heart disease,^16^, ^17^, ^18^, ^19^, ^20^, ^21^, ^22^, ^23^ which in turn can lead to a loss of confidence in guidelines more generally.

It is thus essential that COI are addressed in guideline development processes and publications, to minimize the risk of bias and optimize transparency. Guideline developers need to identify the interests of all contributors that might potentially conflict with the primary interest developing a valid, trustworthy guideline and need to manage any conflicts appropriately. The World Health Organization (WHO),^9^ the Guidelines International Network (GIN),^24^ the American College of Physicians (ACP),^25^ and other organizations have published their COI policies for guidelines in their manuals. However, these policies differ substantially with respect to the types of COI they focus on, recommendations for the management of COI, clarity of presentation, and other key information.^26^, ^27^, ^28^ This variability of content among organizations may also affect guideline contributors' adherence to their COI policies.^17^, ^29^, ^30^, ^31^, ^32^, ^33^

All guidelines need funding to support the guideline development process, including evidence reviews, consensus meetings, writing the guideline, and dissemination and implementation of the guideline. Funders can potentially influence all aspects of guideline development, in a manner similar to the COI of individual contributors. To date, no studies have examined the funding policies of organizations that develop guidelines.

A thorough review of the guideline developers' COI and funding policies is crucial for understanding how these are implemented in guideline development. This study aimed to investigate the characteristics and content of COI and funding policies used by organizations and entities across the globe that develop CPGs and to provide suggestions for further improvement of COI policies in guideline manuals.

## 2 METHODS

### 2.1 Search strategy

Two researchers (ZW and QS) independently searched PubMed from its inception until September 10, 2021, as well as Google, using the search terms ‘guideline methodology’, ‘guideline handbook’, ‘guideline manual’, and their derivatives. The search strategies are presented in detail in the supplement (eMethods). We first screened the titles and abstracts found in the PudMed search, as well as the first ten pages of the search results for each term searched in Google. For potentially relevant records, the full texts were retrieved to decide about inclusion. We also searched the websites of WHO,^34^ GIN,^35^ National Institute for Health and Clinical Excellence (NICE),^36^ and Scottish Intercollegiate Guidelines Network (SIGN).^37^ The websites were searched manually and, if necessary, using the search function until the organization's development manual was found.

### 2.2 Inclusion and exclusion criteria

We included all guideline development manuals, handbooks, and other policy documents that included a policy addressing either COI or funding. Documents were excluded if they were duplicates, had no or insufficient content related to COI or funding, or if the full text was not accessible or was written in a language other than English.

### 2.3 Study selection

We screened the literature using EndNote (version 20.0.1; Clarivate, London, United Kingdom) to facilitate study selection and tracking. After eliminating duplicates, two investigators (ZW and QS) independently screened the titles and abstracts to identify potentially eligible polices and then read the full texts to determine final inclusion. Any discrepancies were resolved by discussion, consulting a third investigator (YX) if necessary.

### 2.4 Definitions related to conflicts of interest and funding

In guideline development, the primary interest of the contributors is to produce recommendations that improve the health and well‐being of the target populations.^9^ Secondary interests include all interests of the contributors other than the primary one(s) that could potentially be related to the recommendations of the guideline.^9^ Below by ‘interests’ we refer to secondary interests only. Interests are not in themselves problematic: only if a (secondary) interest can be expected to unduly affect the contributor's judgment related to the primary interest, does it constitute a COI.

By the terms ‘interests’ and ‘COI’, we refer to interests of the individual contributors to the guideline development, while ‘funding’ means the funding of the guideline project itself.

### 2.5 Data extraction and analysis

Ten investigators (ZW, YZ, YX, PW, MR, YS, HL, YL, SW and QG) were divided into five groups of two. Each group was given a part of the retrieved records. Both investigators in the group independently extracted the following data using a pre‐defined template: 1. Basic descriptive information including organization, country of publication, type of the organization (e.g., society/association, foundation, or government agency), publication year, version, whether the manual included a COI policy, and whether it had a separate COI policy document; 2. Details of the COI policy, particularly the four aspects that a COI policy should usually cover: (1) disclosure of interests (DOI) (the process of declaring secondary interests), (2) assessment of the DOI (the process of evaluating and identifying whether the declared secondary interests constitute COI, and assessing the severity of the COI), (3) management of COI (the process of the measures taken in respect of COI of varying severity that exist), and (4) publication of COI (reporting of the relevant information such as publicly available notice of the COI, access to relevant materials and updates, and other details); and 3. For policy documents that addressed funding, information related to the following aspects: (1) reporting of funding (e.g., the requirements to report the source and amount), (2) management of funding (e.g., the explicit way to use it), and (3) role of the sponsor (e.g., any restrictions to being involved in the process). We determined for each included document whether the aspects mentioned above were addressed or not, and summarized the related contents. More details are presented in eTable 1 in the Supplement.

We used Microsoft Excel 2019 (Microsoft Corporation, Edmond WA, USA) for data collection and descriptive analyses. Dichotomous and categorical variables are presented as absolute numbers and percentages. Data that could not be presented quantitatively are described qualitatively.^38^

## 3 RESULTS

### 3.1 Study selection and characteristics

**Figure 1:**
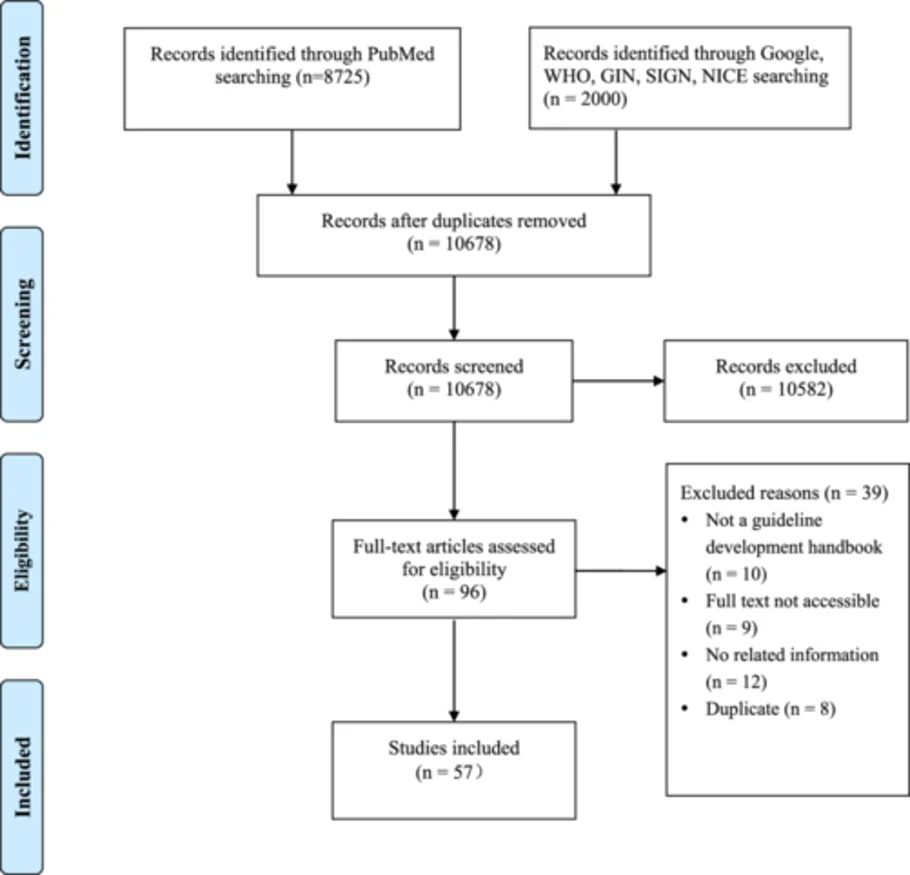
Flow diagram of the literature and web search. COI, conflicts of interest; GIN, Guideline International Network; NICE, National Institute for Health and Care Excellence; SIGN, Scottish Intercollegiate Guidelines Network; WHO, World Health Organization.

We identified 8725 references from PubMed and 2000 records from the Google search. After removing duplicates and screening the titles, abstracts and, when necessary, full‐text documents, 57 guideline manuals were included in our study (Figure 1). Of these 57 manuals, 55 included a COI policy and 28 a funding policy. The manuals were developed by 57 different organizations, 43 (75.4%) of which were societies or associations. Almost half of the manuals (*n* = 26, 45.6%) were from the United States.

### 3.2 Conflicts of interest

Most of the COI policies were contained within the guideline manuals, although 11 (20.0%) of the 55 manuals addressing COI had a separate COI policy document (Table 1). Three of the 55 manuals did not contain a unique, bespoke policy, but referred to other organizations' COI policies instead.^39^, ^40^, ^41^ One of these three, the COI policy document of GIN, almost exclusively referred to policies of other organizations, and we, therefore, excluded it from the quantitative analysis, thus only analyzing 54 COI policies.

**Table 1:** Description of the organizations and their conflict of interest and funding policies.

Organization								
Name	Acronym	Country or region	Type of organization	Publication year	Version	COI policy	Separately published (stand‐alone) COI policy document	Funding policy
Alberta Medical Association	AMA	Canada	Society/Association	2016	NR	Yes	No	Yes
American Academy of Family Physicians	AAFP	USA	Society/Association	2017	NR	Yes	No	Yes
American Academy Neurology	AAN	USA	Society/Association	2017	Revised	Yes	No	Yes
American Academy of Orthopaedic Surgeons	AAOS	USA	Society/Association	2021	Version 4	Yes	No	No
American Academy of Otolaryngology—Head and Neck Surgery Foundation	AAO‐HNSF	USA	Foundation	2013	Version 3	Yes	Yes	Yes
American Academy of Pediatrics	AAP	USA	Society/Association	2019	NR	No	No	Yes
American College of Cardiology Foundation/American Heart Association	ACCF/AHA	USA	Society/Association	2010	NR	Yes	No	Yes
American College of CHEST Physicians	CHEST	USA	Society/Association	NA^a^	NR	Yes	Yes	No
American College of Obstetricians and Gynecologists	ACOG	USA	Society/Association	2021	NR	Yes	Yes	Yes
American College of Occupational and Environmental Medicine	ACOEM	USA	University	2017	Revised	Yes	No	No
American College of Physicians	ACP	USA	Society/Association	2019	NR	Yes	No	No
American College of Rheumatology	ACR	USA	Society/Association	2015	NR	Yes	No	No
American Dental Association	ADA	USA	Society/Association	2013	Update	Yes	No	Yes
American Psychiatric Association	APA	USA	Society/Association	2011	NR	Yes	No	No
American Society for Radiation Oncology	ASTRO	USA	Society/Association	2019	NR	Yes	Yes	No
American Society of Clinical Oncology	ASCO	USA	Society/Association	2019	NR	Yes	No	Yes
American Society of Plastic Surgeons	ASPS	USA	Society/Association	2016	NR	Yes	Yes	Yes
American Thoracic Society	ATS	USA	Society/Association	2015	NR	Yes	Yes	No
American Urological Association	AUA	USA	Society/Association	2015	NR	Yes	No	Yes
Australian National Health and Medical Research Council	NHMRC	Australia	Government	2011	Version 1.1	Yes	No	Yes
British AIDS society	BHIVA	UK	Society/Association	2011	NR	Yes	No	Yes
British Association for Sexual Health and HIV	BASHH	UK	Society/Association	2015	NR	Yes	No	Yes
British Thoracic Society	BTS	UK	Society/Association	2019	NR	Yes	Yes	Yes
Canadian Task Force on Preventive Health Care	CTFPHC	Canada	Government	2014	Version 2	Yes	No	No
Chinese Medical Association	CMA	China	Society/Association	2018	Version 1	Yes	No	Yes
Cincinnati Children's Hospital Medical Center	CCHMC	USA	Hospital	2015	Version 7	Yes	No	No
Council of Europe Publishing	CEP	Europe	Society/Association	2002	NR	No	No	Yes
Endocrine Society	ENDO	USA	Society/Association	NA^a^	NR	Yes	No	No
European Association of Cardiothoracic Surgery	EACTS	Europe	Society/Association	2015	NR	Yes	No	No
European Association of Urology	EAU	Europe	Society/Association	2017	Update	Yes	Yes	No
European Renal Association ‐ European Dialysis and Transplant Association‐ European Renal Best Practice	ERA‐EDTA	Europe	Society/Association	2014	NR	Yes	No	No
European Respiratory Society	ERS	Europe	Society/Association	NA^a^	NR	Yes	Yes	No
European Society for Clinical Nutrition and Metabolism	ESPEN	Europe	Society/Association	2015	NR	Yes	No	Yes
European Society of Cardiology	ESC	Europe	Society/Association	2010	NR	Yes	No	Yes
European Society of Gastrointestinal Endoscopy	ESGE	Europe	Society/Association	2012	NR	Yes	No	No
European Society of Human Reproduction and Embryology	ESHRE	Europe	Society/Association	2019	Version 4.0	Yes	No	Yes
Guidelines and Protocols Advisory Committee‐British Columbia	GPAC	Canada	Government	2017	Revised	Yes	No	No
Guideline International Network	GIN	International	International Organization	NA^a^	NR	Yes	No	Yes
Healthcare Improvement Scotland, Scottish Intercollegiate Guidelines Network	NIS, SIGN	Scotland	Society/Association	2021	Draft 1.0	Yes	No	No
Infectious Diseases Society of America	IDSA	USA	Society/Association	2021	Version 1	Yes	No	Yes
Institute of Medicine	IOM	USA	University	2011	NR	Yes	No	No
Japan Council for Quality Health Care	JCQHR	Japan	Government	2014	Version 1	Yes	No	Yes
National Clinical Effectiveness Committee	NCEC	Ireland	Society/Association	2019	Update	Yes	Yes	No
National EMS Advisory Council and Federal Interagency Committee on EMS	FICEMS, NEMSAC	USA	Government	2012	NR	Yes	No	No
National Health and Medical Research Council	NHMRC	Australia	Government	2017	NR	Yes	No	Yes
National Institute for Health and Care Excellence	NICE	UK	Society/Association	2014	NR	Yes	No	No
New Zealand Guidelines Group	NZGG	New Zealand	Society/Association	2001	NR	Yes	No	No
Royal College of Obstetricians and Gynaecologists	RCOG	UK	Society/Association	2015	Version 3	Yes	No	No
Royal Dutch Society for Physical Therapy	KNGF	Netherlands	Society/Association	2016	Revised	Yes	No	Yes
Scottish Intercollegiate Guidelines Network	SIGN	Scotland	Society/Association	2019	Revised	Yes	No	Yes
Society for Vascular Surgery	SVS	USA	Society/Association	2011	NR	Yes	No	No
Society of Interventional Radiology	SIR	USA	Society/Association	NA^a^	NR	Yes	Yes	No
The Saudi Center for Evidence Based Health Care	SCEBHC	Saudi Arabia	Government	2014	Version 1.0	Yes	No	No
U.S. Preventive Services Task Force	USPSTF	USA	Society/Association	2021	NR	Yes	No	No
UK Renal Association	UKRA	UK	Society/Association	2011	NR	Yes	No	Yes
World Health Organization	WHO	International	International Organization	2014	Version 2	Yes	No	Yes
Estonian Health Insurance Fund, University of Tartu and the Guideline Advisory Board, Republic of Estonia Ministry of Social Affairs	EHIF, University of Tartu, REMSA	Estonia	Government	2020	Adapted	Yes	No	Yes

Abbreviations: NA, Not applicable; NR, Not reported.

^a^ Website.

#### 3.2.1 Contents of the policies on conflicts of interest

**Figure 2:**
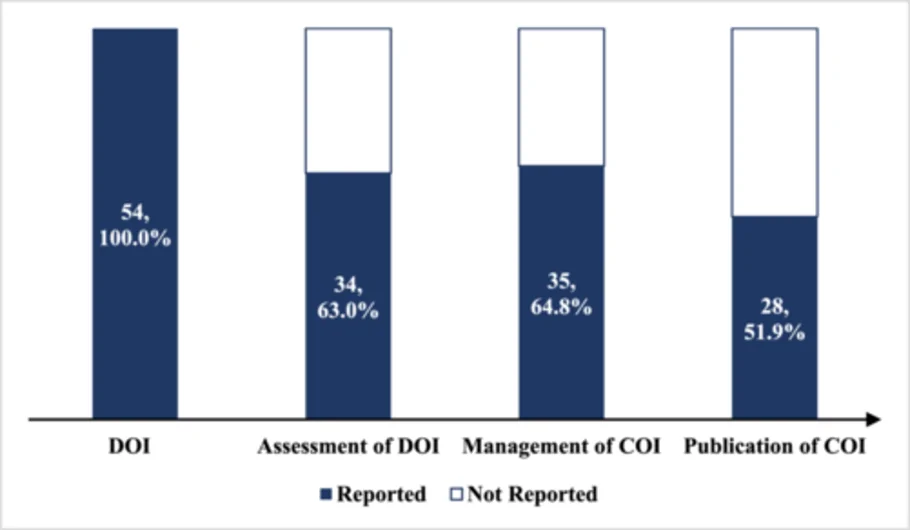
Reporting rates of different aspects of conflicts of interest policies. COI, conflicts of interest; DOI, disclosure(s) of interests.

Most COI policies consisted of the following four sections: disclosure of the interests of contributors, assessment of disclosures, management of COI and publication of COI. All 54 (100%) COI policies addressed the disclosure of interests, 34 (63.0%) the assessment of disclosures, 35 (64.8%) the management of COI, and 28 (51.9%) the publication of COI (Figure 2, Table 2).

**Table 2:** Items included in conflicts of interest policies.

Item	No (%)
Disclosure of interests	*N* = 54
*Who needs to disclose interests*	
Reported	52 (96.3)
Not reported	2 (3.7)
*How to disclose the interests*	
Reported	43 (79.6)
Using declaration of interest forms	30 (55.5)
Using other approaches^a^	13 (24.1)
Not reported	11 (20.4)
*Who should collect disclosures*	
Reported	19 (35.2)
Not reported	35 (64.8)
*What needs to be disclosed*	
Reported	18 (33.3)
Not reported	36 (66.7)
*How should COI be categorized*	
Reported	20 (37.0)
Financial COI and nonfinancial or intellectual COI	18 (33.3)
Other categorization^b^	2 (3.7)
Not reported	34 (63.0)
*Updating DOI*	
Reported	19 (35.2)
Annually	6 (11.1)
If any changes occur or new interests emerge	3 (5.6)
At least annually	3 (5.6)
Others^c^	7 (13.0)
Not reported	35 (64.8)
**Assessment of DOI**	***N*** = **34**
*Who should assess the DOI*	
Reported	34 (100.0)
Not reported	0 (0.0)
*Categorization of COI by severity*	
Yes	16 (47.1)
No	18 (52.9)
**Management of COI**	***N*** = **35**
*Who should manage the COI*	
Reported	26 (74.3)
Not reported	9 (25.7)
*How should COI be managed*	
Reported	29 (82.9)
By category	18 (51.4)
Not reported	6 (17.1)
**Publication of COI**	***N*** = **28**
*How can COI be accessed*	
Reported	28 (100.0)
Published within the guideline	19 (67.9)
Available on the organization's website	7 (2.05.0)
Published within the guideline and available on the website	1 (3.6)
Files stored at the organization's office	1 (3.6)
Not reported	0 (0.0)
*The guideline should report the evaluation and management of COI*	
Reported	6 (21.4)
Not reported	22 (78.6)

Abbreviations: COI, conflict(s) of interest; DOI, disclosure of interests.

^a^ American Urological Association (AUA) interactive website (*n* = 1); declare competing interests both verbally and in writing (*n* = 1); disclosure statement (*n* = 2); relationships are disclosed in writing or online (*n* = 1); sign a declaration regarding any competing interests (*n* = 1); structured electronic format (*n* = 1); the Declaration of Affiliations and Interests Form and Checklist (*n* = 1).

^b^ Personal and Nonpersonal Interests (*n* = 1); Real COI, potential COI and perceived (or apparent) COI (*n* = 1).

^c^ At each meeting of the guideline development group (GDG) (*n* = 1); before each Clinical Guidelines Committee meeting and each year (*n* = 1); before each panel meeting (*n* = 1); before, or at, the first meeting of the GDG and on an annual basis thereafter for the period that the GDG is active (*n* = 1); during each conference call, at each in‐person meeting, and before publication (*n* = 1); during the first year (*n* = 1).

#### 3.2.2 Definition of ‘conflict of interest’

Of the 54 manuals that addressed disclosure of interests, only 19 (35.2%) explicitly defined the concept of COI (Table 3). The manuals of the American Academy of Otolaryngology‐Head and Neck Surgery Foundation and the WHO^9^ followed or referred to either the 2009^38^ or the 2011^1^ definition of COI from the US Institute of Medicine (IOM). Fifty‐two (96.3%) manuals specified the persons required to disclose secondary interests.

**Table 3:** Definitions of conflicts of interest used by organizations.

Organization^a^	Definition of COI
AAFP	A COI has been defined as a set of conditions in which professional judgment concerning a primary interest (guideline recommendations), is unduly influenced by a secondary interest (financial or intellectual interests).^28^, ^38^
AAN	A person has a conflict of interest in a situation where their professional responsibilities, such as patient welfare or their Academy role or service, are or may be compromised by the influence of financial gain or a relationship with another entity that confers personal benefit.
AAO‐HNSF	Conflict of interest (COI) may be defined as a set of circumstances that creates a risk that professional judgment or actions regarding a primary interest will be unduly influenced by a secondary interest.^1^
ACCF/AHA	The ACCF and AHA prefer the term Relationships with Industry (RWI) and Other Entities as opposed to the term Conflict of Interest (COI). RWI, by definition, does NOT necessarily imply a conflict. When all relationships are disclosed with the appropriate detail regarding category and amount, and managed appropriately for building consensus and voting, the ACCF/AHA believes that potential bias can be avoided and the final published document is strengthened since the necessary expertise is accessible.
ACOG	A conflict of interest may arise when a Covered Individual has some interest or obligation that has the potential to create divided loyalty on the part of the Covered Individual's loyalty between ACOG and some other organization or cause. A conflict of interest may arise from a transaction between ACOG and a third party, or from a Covered Individual's volunteer, paid, or other financial relationship with a third party, which may compromise a Covered Individual's ability to provide unbiased judgment and undivided loyalty to ACOG.
ACP	Any declared interest that may affect or be perceived to affect objectivity and independence.
ASCO	ASCO defines the following relationships as conflicts of interest: 1. Research funding from an affected Company, paid to the individual or his or her practice or institution if: a. research payments are made directly from the affected Company to the individual; b. the individual's salary is supported (in whole or part) through a research grant from an affected Company; c. the individual is a national or overall principal investigator for a study funded by an affected Company; d. the individual is a member of a steering committee of a study that does not have a principal investigator.^9^ 2. Compensation (including honoraria) from any one affected Company that equals, in aggregate, $5,00010 or more in a calendar year. a. This includes fees and honoraria for leadership positions, consulting activities, speaking engagements, expert testimony, and patent or other licensing fees. b. This excludes any compensation provided under any of the circumstances described in Section IV.a.
ASPS	A conflict of interest is defined as an interest held by a member that could influence the member or be perceived as influencing the member to act contrary to the interests of the Society or the Foundation and for the member's own personal benefit or for the benefit of an immediate family member or business associate.
CHEST	Conflict of Interest (COI) refers to any relationship or other set of known circumstances that has the potential to bias, or that might be reasonably perceived by others to bias, an individual s judgment, conduct, or other work.
EACTS	A COI exists when an individual s personal interests, e.g., direct and indirect financial or intellectual, have the potential to compete with or influence behaviour related to the individual s professional interests or obligations, i.e., evaluating the evidence and drafting recommendations for clinical practice guidelines.^39^
EAU	A COI is defined as a set of conditions in which an individual s objectivity, professional judgement, professional integrity and/or ability to perform his/her responsibilities may be unduly influenced by a secondary interests (such as personal or financial gain), or which may be perceived by others to bias an individual s judgement, conduct, or work.
GPAC	The definition of a conflict of interest includes a situation in which personal, occupational or financial considerations may influence a member's decisions or affect the objectivity or fairness of a member of GPAC.
IOM	A set of circumstances that creates a risk that professional judgment or actions regarding a primary interest will be unduly influenced by a secondary interest.^34^ A divergence between an individual's private interests and his or her professional obligations such that an independent observer might reasonably question whether the individual's professional actions or decisions are motivated by personal gain, such as financial, academic advancement, clinical revenue streams, or community standing. A financial or intellectual relationship that may impact an individual's ability to approach a scientific question with an open mind (Schünemann et al., 2009, p. 565).^10^
NCEC	The Institute of Medicine (2009, p. 46) define COI as 'a set of circumstance that creates a risk that professional judgement or actions regarding a primary interest will be unduly influenced by a secondary interest.^34^
NICE	There is a conflict of interest when a reasonable person would consider that an individual's ability to apply judgement or act in the work of NICE is, or could be perceived to be, impaired or influenced by one of their interests.
NZGG	Declaration of Competing Interests/Conflicts of Interest – describe any funding, sponsorship, employment relationships, memberships or affiliations that may be perceived to have had some influence on group participants.
SIGN	Competing interests are defined as any interest of the person, their partners or close relatives (personal) or their department/employer/business (nonpersonal) which may potentially influence the content, including recommendations, of SIGN guidelines. For clarity, the interest of partners or close relatives is restricted to employment in, or shareholdings in, healthcare organizations.
WHO	A conflict of interest is a set of circumstances that creates a risk that professional judgment or actions regarding a primary interest will be unduly influenced by a secondary interest.^1^ The Declaration of Interests form at WHO^40^ contains a similar definition: [A conflict of interest is] any interest declared by an expert that may affect or reasonably be perceived to affect the expert s objectivity and independence in providing advice to WHO.
EHIF, University of Tartu, REMSA	A COI is ‘a divergence between an individual's private interests and his or her professional obligations such that an independent observer might reasonably question whether the individual's professional actions or decisions are motivated by personal gain, such as direct financial, academic advancement, clinical revenue streams, or community standing’.^10^

*Note*: Definitions are direct quotations from the manual or policy.

Abbreviation: COI, conflict(s) of interest.

^a^ See Table 1 for the full names of the organizations.

#### 3.2.3 Disclosure of interests

The WHO manual^9^ stated that all members of the guideline development group, individuals who prepare systematic reviews and evidence profiles, guideline methodologists, technical writers, and any other experts who participate in the process in an individual capacity should disclose their relevant interests. Other manuals varied greatly with respect to who was required to provide a disclosure (or declaration) of interests. The requirements in manuals of other agencies are presented in eTable 2 of the Supplement.

DOI forms were the most common format for the identification of secondary interests in the manuals (*n* = 43, 79.6%). Nineteen (35.2%) manuals reported who should collect the disclosures. Eighteen (33.3%) manuals reported the specific interests that needed to be disclosed: for example, the WHO manual^9^ stated in Section 6.6: ‘What interests need to be disclosed?’ Twenty (37.0%) manuals categorized the types of interests to be disclosed: financial versus nonfinancial or intellectual COI was the most common form of categorization (*n* = 18, 33.3%). Twelve manuals explicitly reported the definition, scope or specific content of financial COI and nonfinancial or intellectual COI. Possessing equities, stock or shares (*n* = 11, 91.7%) were the most frequently mentioned types of financial COI. Membership in an advisory board, committee, organization or advocacy group (*n* = 8, 66.7%) was the most commonly mentioned type of nonfinancial or intellectual COI (see eTable 3 and eTable 4 in the Supplement). Nineteen (35.2%) manuals required updating of DOI and specified a frequency for updates; annual updates were the most common (*n* = 6, 11.1%; Table 2).

A total of 13 policies addressed the monetary value of the secondary interest. Eleven of these policies specified a threshold at which the secondary interest needed to be disclosed, and the remaining two policies used ranges of amount (e.g., $100–$1,000, $1,001–$5,000) in the disclosure form (see eTable 5 in the Supplement).

All 34 manuals that addressed DOI reported who should perform the review and assess them. According to the WHO manual,^9^ the DOI should be evaluated by the responsible officer for the guideline and his/her director, with consultation with the ethics office, if needed. The COI policy document of SIGN^42^ requires the DOI to be evaluated by the chairpersons of the SIGN council, SIGN Executive Senior Management Team, and SIGN subcommittees, among others (Table 2). The DOI assessors specified in all manuals are listed in eTable 6 in the Supplement.

#### 3.2.4 Management of conflicts of interest

**Figure 3:**
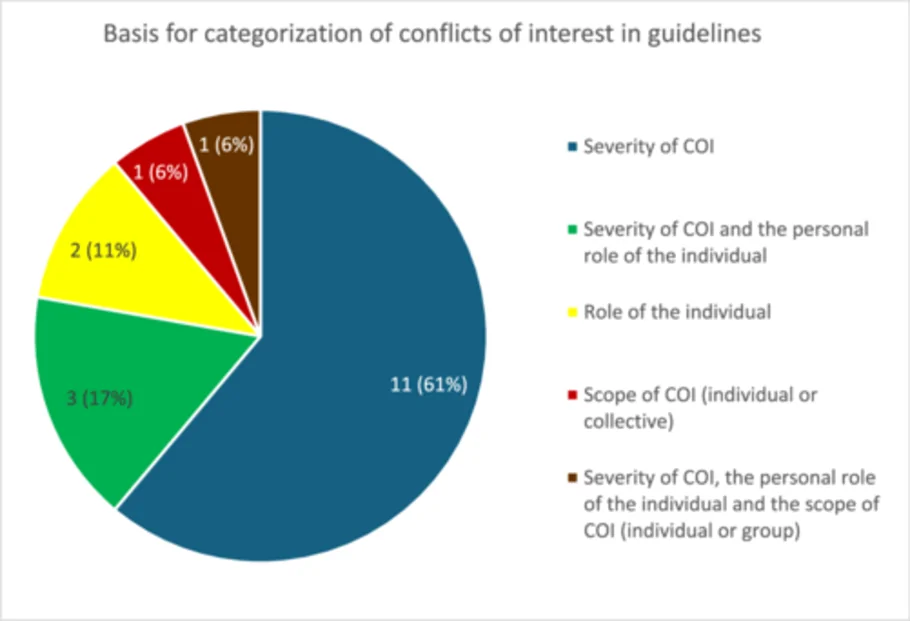
Basis for categorization of conflicts of interest in guidelines (*N* = 18 manuals).

Among the 35 manuals that mentioned the management of COI, 26 (74.3%) specified who should be responsible for determining and implementing a management plan. The WHO manual^9^ stipulates that the WHO responsible technical officer should manage the COI in consultation with the director of the technical unit, while SIGN's COI policy^43^ stipulates that COI are governed by the SIGN Senior Management Team. The recommendations on who should manage COI are listed in eTable 7 of the Supplement. Twenty‐nine (82.4%) manuals reported specific management methods, of which 18 suggested categorizing the COI to facilitate management. The most common approach was to manage COI based on their severity, leading to unrestricted participation, partial restriction, or complete exclusion from the guideline development process (*n* = 11, 61.1%; Table 2, Figure 3, Supplement eTable 8).

#### 3.2.5 Publication of conflicts of interest

All 28 manuals addressing the publication of COI indicated how COI statements could be publicly accessed. Publication of the COI along with the guidelines was the most commonly suggested method (*n* = 19, 67.9%). Only six (21.4%) manuals stipulated that guidelines should report the COI assessment and management processes.

### 3.3 Funding

Of the 28 manuals containing guidance on funding for a guideline, eight (28.6%) required the source(s) of the funding to be reported. Funding could be reported in different sections of the guideline,^9^, ^44^, ^45^, ^46^, ^47^ such as in the methods section,^48^ in the Statement of Interest or Financial Sponsor section,^49^ or already in the protocol.^9^ One manual required reporting of details of the funding sources,^9^ and one the amount and percentage of funding by source.^50^ None of the manuals required the guideline to report how funding was explicitly used in guideline development. Four (14.3%) manuals reported the types of funding the guidelines were permitted to receive, and eight (28.6%) manuals declared the types of funding sources that guidelines should not accept. Only five (17.9%) manuals required the guidelines to limit the authority of funders in the guideline development process. However, 14 (50.0%) manuals required an explicit statement that the views or interests of funders did not influence the final recommendations in the guideline (Table 4).

**Table 4:** Items included in policies related to funding.

Type	No (%)
**Reporting of funding**	
*Requirement to report on the source of funding*	
Reported	8 (28.6)
Not reported	20 (71.4)
*Requirement to report funding details*	
Reported	2 (7.1)
Not reported	26 (92.9)
*Requirement to report on how funding is used*	
Reported	0 (0.0)
Not reported	28 (100.0)
*Requirement to report on whether funding affects guideline recommendations*	
Reported	0 (0.0)
Not reported	28 (100.0)
**Management of funding**	
*Which type of funding should not be accepted for guideline development*	
Reported	14 (50.0)
Not reported	14 (50.0)
*Which type of funding can be accepted for guideline development*	
Reported	8 (28.6)
Not reported	20 (71.4)
*Who should manage the funding*	
Reported	4 (14.3)
Not reported	24 (85.7)
**Role of the sponsor**	
*Should the authority of sponsors be limited in the development of guidelines*	
Reported	5 (17.9)
Not reported	23 (82.1)
*Who should judge whether funders can participate in guideline development*	
Reported	1 (3.6)
Not reported	27 (96.4)

## 4 DISCUSSION

All guideline COI policies we identified reported requirements for the disclosure of relevant interests, and most manuals addressed the review and assessment of the disclosures, how the identified COI should be managed, and the publication of COI. However, details of the processes, procedures and requirements related to other aspects of COI varied substantially across the manuals. Half of the manuals included a policy on funding disclosure, addressing mainly the sources of funding. Half of the funding policies had a requirement that the sponsor's views or opinions should not influence the final recommendations. Some manuals explicitly stated the types of funding that guideline developers should not accept.

With the rapid advancement of evidence‐based medicine in the last decade, significant progress has been made in the methodology of guideline development. In response to the challenges that COI pose for guideline development, the US IOM published a report on the characteristics of trustworthy guidelines in 2011 to guide the development, reporting and implementation of guidelines. This report drew particular attention to COI, which should be considered from the beginning throughout the entire guideline development process.^1^ Thus, a comprehensive policy addressing COI of all contributors and a policy concerning funders are prerequisites for ensuring that guidelines are scientifically correct and recommendations are valid, transparent and trustworthy. Based on our findings, we make the following suggestions.

First, a clear, specific definition of COI should be provided in all COI policies: this can facilitate the disclosure of relevant interests by all guideline contributors. We found that only about one‐third of policies provided a clear definition of COI, which may be one of the main reasons for incomplete disclosure of COI in guidelines.^31^, ^33^ However, even when a definition of COI is provided, the definitions vary and are often vague and difficult to interpret.^27^, ^51^ In addition, the clarity and comprehensiveness of the DOI form need to be enhanced, and details of the COI policy such as the relevant types of interests, how they are categorized, and thresholds for disclosing financial COI, need to be made clear. Schedules for updating disclosures also need to be augmented. Improving the clarity of the COI policy can facilitate compliance with the policy when developing the guideline, and thus help to achieve a more transparent, valid, and trustworthy guideline.^29^, ^30^

Second, all guideline development organizations should establish a COI management committee, whose members should be free of COI. Most of the manuals specified the persons who should review the COI. It comes without saying that COI assessors themselves need to fulfil stringent requirements. Guideline development organizations should clearly specify the criteria for assessing the DOI, and for determining if a COI exists and its potential significance. Such pre‐specification facilitates transparency, adherence to the policy, and consistency in reporting and management. The management of COI should be based on the potential (real or perceived) ability of the interest to impact the recommendations in the guideline. Depending on the level of the COI, the respective contributor may be permitted participation in the entire guideline development, may be completely excluded, or may be permitted partial or restricted participation (i.e., participate only in questions unrelated to the COI). We identified only 18 COI policies that articulate specific management approaches depending on the significance of the COI. Thus, there is much room for improvement in this important aspect of COI policies: we think policies need to provide as many details as possible about the specific management of each type of COI.^2^

Third, all guideline development organizations should be encouraged to make their COI policies, a summary of the DOI of each contributor, and the assessment and management of any COIs publicly available. This facilitates transparency and helps to ensure the guidelines are credible. If details of the processes, methods and DOI are limited by the layout of the guideline, a link to an online document can be provided on the guideline organization's website.

Guideline funding is an important source of COI. The suggestions made above for individual contributors also apply to policies regarding the funders for guidelines. Funding from entities with an interest in achieving specific recommendations (e.g., supporting a particular drug or technology they are involved with) should not be accepted. Even if such a funder is reported as ‘hands‐off’, the credibility of the guideline may be compromised.

At least three previous reviews have so far evaluated COI policies. Norris and colleagues^28^ analyzed the COI policies of organizations with at least five guidelines listed in the US National Guidelines Clearinghouse (NGC) between January 2009 and November 2010. Morciano et al.^27^ analyzed COI policies published between September 2014 and June 2015, covering a wide range of health topics. Brems et al.^26^ analyzed the COI policies of organizations that published five or more guidelines between January 2018 and December 2019. These previous reviews have some differences in their approaches which makes it challenging to draw relevant conclusions from them. The work of Norris et al. was only based on the NGC, which is not representative of all guidelines published globally, and presents the information from a source that does not exist anymore (NGC was inactivated in 2018^52^). The work by Morciano et al., on the other hand, included only organizations that produce general health‐related guidelines, and therefore excluded professional organizations with a narrower disease or other focus. Moreover, the studies by Norris et al.^28^ and Brems et al.^26^ were limited to COI policies published in English and compared the COI policies to IOM standards, while Morciano et al.^27^ included three additional languages. Thus, these studies examined samples of COI policies that may not be representative of the policies of all CPG‐producing organizations. In addition, as evidence‐based guideline development methods have evolved, many guideline development organizations have updated their COI policies or have begun to develop them. Finally, none of the prior reviews examined policies related to funding. Our study thus represents the most up‐to‐date comprehensive examination of COI and funding policies for CPGs. These results can help organizations with existing COI and funding policies to update them, while providing a reference for other organizations to develop their own policies.

Recently, a checklist for reporting on COI and funding in guidelines and guideline developers' organizational policies, the RIGHT‐COI&F Statement, was published.^53^ We hope that this new tool can help the developer organizations in formulating their policy documents rigorously and assure that all relevant details are covered. In addition, the RIGHT‐COI&F statement can be used as an assessment tool to evaluate the compliance of guidelines and policy documents with universal standards.

This study has several limitations. First, we included only manuals from organizations whose COI or funding policies were publicly accessible. Some other organizations may have policies that we were unable to identify. Second, we did not analyze the differences in the policies in detail, for example between different regions or organization types. It would be challenging, however, to perform a thorough comparison because of the heterogeneity among the organizations: for example, the need for a funding policy depends on the financing of the organization itself. Third, we did not contact any of the guideline development organizations to confirm whether the publicly accessible versions of their COI or funding policies were up to date, or to identify missing or additional information. We also did not examine the compliance of guidelines with the COI or funding policies of the respective developer organizations; nor did we study the actual impact of the policies on the guideline‐development process, collection of DOI, or management and reporting of COI. We acknowledge that a rigorous policy alone is not yet sufficient if it is not properly implemented, and the adherence of guidelines to policies should be investigated. Finally, we included only COI and funding policies published in English, which may limit the generalizability and applicability of our findings to guidelines published in other languages. Future research, particularly since the release of the RIGHT‐COI&F guidance,^53^ should also focus on a quantitative analysis of the compliance of guidelines and policy documents to these recommendations, and explore the reasons behind suboptimal coverage in more detail.

## 5 CONCLUSION

Although most guideline development organizations emphasize the importance of transparent declaration of relevant interests and management of any COI, many policies lack key elements such as a precise definition of COI, and detailed descriptions of the key steps in COI management, what constitutes appropriate funding, and how the sources, use and potential influence of funding should be reported. Unclear and incomplete COI policies may potentially confuse interest‐holders such as end‐users and policymakers, undermine public confidence in the guidelines, and most importantly, lead to biased or invalid recommendations that can harm individuals and public health. Future guidelines should be based on comprehensive COI and funding policies that are rigorously implemented, to ensure that the recommendations are clear, valid, and credible.

## AUTHOR CONTRIBUTIONS

Yangqin Xun, Qiangqiang Guo and Zijun Wang had full access to all of the data in the study and take responsibility for the integrity of the data and the accuracy of the data analysis. Yangqin Xun, Zijun Wang, Susan L Norris, Yaolong Chen, Janne Estill conceived and designed the study, and conducted data analysis. Yangqin Xun, Qiangqiang Guo, Zijun Wang, Ying Zhu, Nan Yang, Yajia Sun, Shouyuan Wu, Mengjuan Ren, Ping Wang, Hui Liu, Hui Lan, Yunlan Liu and Qianling Shi collected the data; Yangqin Xun and Zijun Wang drafted the manuscript; Akihiko Ozaki, Susan L Norris, Ivan D. Florez, Joseph L. Mathew, Myeong Soo Lee revised the manuscript; Yaolong Chen and Janne Estill supervised this study.

## CONFLICT OF INTEREST STATEMENT

YX is funded by China Scholarship Council (No. 202106180043). H.L. is funded by China Scholarship Council (No. 202206180006); N.Y. is funded by China Scholarship Council (No. 202206180007). Other authors have nothing to declare.

## ETHICS STATEMENT

The authors have nothing to report.

## Supporting information

Supporting information.

## Data Availability

Research data are not shared.
