# Supplementary material for: A cross‐sectional review of policies on conflicts of interest and funding in the development manuals of practice guidelines
Source: Clin Public Health Guidel. 2025 Apr 10;2(2):e70020. doi: 10.1002/gin2.70020 (PMC13563319; doi:10.1002/gin2.70020)
Supplement: Supplementary file 1 [file GIN2-2-e70020-s001.docx]

**eMethods: Search Strategy**

**Google**

We searched for each of the following terms separately, and assessed records from the first 10 pages for eligibility.

1 guideline methodolog*

2 guideline handbook3 guideline manual

4 guideline toolkit

5 guidance methodolog*6 guidance handbook

7 guidance manual

8 guidance toolkit

9 consensus methodolog*

10 consensus handbook

11 consensus manual

12 consensus toolkit

13 recommendation methodolog*

14 recommendation handbook

15 recommendation manual

16 recommendation toolkit

17 standard methodolog*

18 standard handbook

19 standard manual

20 standard toolkit

**PubMed**

#1 Clinical Practice Guideline*[tw]
#2 Clinical guideline*[tiab]
#3 Guideline*[ti]
#4 guidance*[ti]
#5 consensus[ti]
#6 recommendation*[ti]
#7 OR#1-#6
#8 methodolog*[tiab]
#9 handbook*[tiab]
#10 manual*[tiab]
#11 toolkit*[tiab]
#12 OR#8-#11
#13 #7 AND #12
#14 Practice Guideline[pt]
#15 #13 NOT #14

**eTable 1.** **Template for data extraction and explanation of all items to be extracted**

| 1. Basic descriptive information | | |
| --- | --- | --- |
|  | organization, country of publication, type of the organization (e.g. society/association, foundation, government agency, etc.), publication year, version, whether it had an additional COI policy document, and whether it had an independent section about COI. | |
| 2. Contents related to the COI policy | | |
|  | (1) disclosure of interests | whether the disclosure of COI was addressed, whether an explicit definition of COI was given, who should disclose COI, how COI should be disclosed, who should collect COI information, which types of COI were addressed, and whether the schedule for COI disclosure and update was specified. |
|  | (2) assessment of DOI | whether the review of COI was addressed, who should review the COI, and whether the COI were categorized by their severity. |
|  | (3) management of COI | whether the management of COI was addressed, who should manage the COI, and the management methods. |
|  | (4) publication of COI | whether the publication of COI was addressed, how the COI should be published and how the full information related to COIs should be made obtainable, and whether the guideline should be required to report the COI management and evaluation process. |
| 3. Contents related to funding | | |
|  | (1) reporting of funding | whether receiving any funding should be reported, whether the source of funding should be reported, whether additional details of the source of funding (such as the grant number) should be reported, what contents related to funding need to be reported, whether it should be reported how the funding was used and what the impact of funding was, and whether the manual requested to add a statement that the views or opinions from funders did not affect the final recommendations. |
|  | (2) management of funding | which kind of funding the guideline should not accept, which kind of funding the guideline can accept, and who should manage the funding. |
|  | (3) role of the sponsor | whether the authority of funders in guideline development should be restricted, and who should decide whether the funders can participate in the guideline development. |

**eTable 2.** **Organizations’ requirements for who should provide disclosure of interests**

| **Organization*** | **Who provides disclosure of interests** |
| --- | --- |
| AAFP | Individuals being considered for membership |
| AAN | Prospective panel members for AAN guideline projects |
| AAO-HNSF | Guideline development group members and expert reviewers or advisers who are not officially part of the development group |
| AAOS | Applicants |
| ACCF/AHA | those involved in the writing effort (i.e., authors, external peer reviewers, and Task Force on Practice Guidelines) |
| ACOEM | All panel members |
| ACOG | Each committee member |
| ACP | Any potential participant (defined in the Table) in work related to ACP clinical guidelines. This includes CGC members, members of the CGC's public panel, persons involved in the supporting systematic reviews commissioned by ACP, ACP staff, and any other guest attending a CGC meeting or conference call. |
| ACR | Each guideline project development group member and others who contribute intellectually to the guideline development |
| ADA | Individuals who are invited to serve on an expert panel |
| AMA | Each committee member |
| APA | Candidates for the SCPG (Steering Committee on Practice Guidelines) and work groups before their appointment by the APA President. |
| ASCO | Individuals involved in drafting, reviewing, and approving guideline recommendations |
| ASPS | Each officer, director, trustee, committee chair, committee member, task force chair, task force member, the editor and members of the editorial boards of the Plastic and Reconstructive Surgery Journal and Plastic and Reconstructive Surgery-Global Open and others holding leadership positions in the Society and Foundation |
| ASTRO | All individuals involved in drafting, reviewing and approving ASTRO Papers |
| ATS | All individuals who are invited to participate in a guideline development panel |
| AUA | Panel members |
| BASHH | Writing committee and clinical effectiveness group members |
| BHIVA | All members of BHIVA guidelines writing groups as well as any expert external peer reviewers and literature searchers |
| BTS | Each member of the guideline development group |
| CCHMC | Group / Team members (multidisciplinary team and content reviewers/ consultants) |
| CHEST | All potential panelists |
| CMA | All invited members to participate in guideline constitution/amendment |
| CTFPHC | A potential participant (such as a peer reviewer, clinical expert, or review team member) becomes engaged in a CTFPHC-led initiative CTFPHC members |
| EAU | All those participating in EAU clinical Guidelines development |
| ENDO | To be considered for membership of a Writing Committee, nominees |
| ERA-EDTA | Potential guideline development group membership |
| ERS | All ERS officers and members of ERS committees, working parties and subcommittees |
| ESC | All the proposed members of a Task Force |
| ESHRE | All guideline development group chairpersons and members |
| ESPEN | All contributors |
| FICEMS, NEMSAC | All parties |
| GPAC | Committee members |
| IDSA | Chairs and panel members as well as SPGC committee members, IDSA BOD members, guideline reviewers, or any other participants involved in the guideline development process (All participants) |
| JCQHR | All candidates potentially engaged in clinical practice guideline development |
| KNGF | Work and review group members |
| NCEC | The members of the guideline development group and external reviewers |
| NHMRC | All guideline development group members |
| NICE | All committee members, including the chair, and anyone who has direct input into the guideline |
| NIS, SIGN | All guideline development group members |
| NZGG | The members of the group |
| RCOG | All those involved in the development of the Green-top Guidelines, including the Guidelines Committee, Guidelines Co-Chairs, guideline developers, peer reviewers and other external reviewers |
| SCEBHC | Nominated Panel members |
| SIGN | All persons involved in the development of SIGN guidelines |
| SIR | All prospective panel chairs, co-chairs and panel members |
| SVS | Committee members |
| UKRA | All co-authors |
| USPSTF | Task Force members |
| WHO | All members of the guideline development group, individuals who prepare systematic reviews and evidence profiles, guideline methodologists, the technical writer and any other expert who participates in the process in an individual capacity. |
| EHIF, University of Tartu, REMSA | Each Panel member, including the Chair, the nominated Guideline Unit member, and consultant (if involved) |

*See the full names of the organizations in Table 1 of the main text

**eTable 3.** **Scope of financial conflicts of interest**

| **Organization*** | **Type of financial COI** | | | | | | | |  |
| --- | --- | --- | --- | --- | --- | --- | --- | --- | --- |
|  | Paid employment | Paid consultancy or speaking engagement, honoraria, advisory role, board membership | Research grant or salary support | Individual company income or economic advancement of groups | Patents or royalties; f. Equity/stock or shares | Equity/stock or shares | Gifts | Others (e.g., travel grants, attending educational events) |  |
| AAFP | N | Y | Y | Y | N | Y | N | Y |  |
| AAO-HNSF | Y | Y | Y | N | Y | Y | Y | Y |  |
| ACP | Y | Y | Y | Y | Y | Y | N | Y |  |
| CHEST | N | N | N | N | Y | Y | N | Y |  |
| EAU | Y | Y | Y | Y | Y | Y | N | Y |  |
| JCQHR | Y | N | Y | Y | N | N | N | Y |  |
| NCEC | N | Y | N | Y | N | Y | N | N |  |
| NICE | Y | Y | N | Y | Y | Y | N | N |  |
| SIGN | Y | Y | N | Y | N | Y | Y | N |  |
| USPSTF | N | Y | Y | Y | Y | Y | N | Y |  |
| WHO | Y | Y | Y | N | Y | Y | N | Y |  |
| **"Y" responses: n (%)** | **7 (63.6)** | **9 (81.8)** | **7 (63.6)** | **8 (72.7)** | **7 (63.6)** | **10 (90.9)** | **2 (18.2)** | **8 (72.7)** |  |
| Y: Yes; N: No; NR: Not reported  *See the full names of the organizations in Table 1 of the main text | | | | | | | | | |

**eTable 4.** **Scope of non-financial or intellectual conflicts of interest**

| **Organization*** | **Type of non-financial or intellectual COI** | | | | | | |  |
| --- | --- | --- | --- | --- | --- | --- | --- | --- |
|  | Development of related guidelines and standards, educational material | Clinical specialty or preferences | Academic advancement | Publications, trials, systematic reviews | Having personal convictions (political, religious, ideological or other) related to the guideline topic that may interfere with an unbiased evidence review or recommendation process | Membership of advisory boards, committees, organizations, advocacy groups | Others (e.g., expert testimony, public statement, speech, lecture, opinion leader, reputational risks) |  |
| AAFP | N | N | N | Y | N | Y | Y |  |
| AAO-HNSF | Y | N | N | Y | Y | Y | Y |  |
| ACP | N | Y | Y | N | N | N | N |  |
| CHEST | NR | NR | NR | NR | NR | NR | NR |  |
| EAU | N | Y | N | Y | Y | Y | N |  |
| JCQHR | N | Y | Y | N | N | Y | Y |  |
| NCEC | N | N | N | Y | N | Y | N |  |
| NICE | N | N | N | Y | N | Y | Y |  |
| SIGN | N | N | N | N | N | Y | Y |  |
| USPSTF | N | Y | N | N | N | Y | Y |  |
| WHO | N | Y | Y | Y | N | N | Y |  |
| **"Y" responses: n (%)** | **1 (9.1)** | **5 (45.5)** | **3 (27.3)** | **6 (54.5)** | **2 (18.2)** | **8 (72.7)** | **7 (63.6)** |  |
| Y: Yes; N: No; NR: Not reported  *See the full names of the organizations in Table 1 of the main text | | | | | | | | |

| **eTable 5.** **Thresholds for disclosing secondary interests** | |
| --- | --- |
| **Organization*** | **Threshold** |
| AAN | Holding significant ownership interest (shares greater than $50,000 in value or an equity interest in a privately held company greater than 5 percent) in an affected company |
| ACCF/AHA | A person is deemed to have a significant interest in a business if the interest represents ownership of 5% or more of the voting stock or share of the business entity, or ownership of $10,000 or more of the fair market value of the business entity, or if funds received by the person from the business entity exceed 5% of the person’s gross income for the previous year. |
| ACOEM | In addition to sending a copy of your CV, please specify the names and nature of the relationship for the questions below (only include relationships that are or potentially could be worth more than $10,000 during the course of panel participation). When appropriate, you may indicate “see CV” but please be specific (e.g., “see CV, page 4). |
| ACOG | At least annually, each committee member must complete and submit a disclosure form detailing all financial (in excess of $100), business, or other interest in any company, organization, service, product, or other concerns that might affect or be affected by ACOG |
| APA | In accordance with APA policy, members of the SCPG including the Executive Committee must make annual disclosures and may not receive >$10,000 per year from industry work excluding grants. |
| ASCO | Compensation (including honoraria) from any one affected Company that equals, in aggregate, $5,00010 or more in a calendar year; This dollar value may be updated periodically to keep pace with current standards. |
| Cincinnati Children's Hospital Medical Center | Financial Interest does not include: Equity interests that, when aggregated for the Covered Individual and his/her family, do not exceed $5,000 in fair market value and do not represent a five (5) percent or greater ownership interest in a single entity |
| CTFPHC | To avoid potential financial conflicts of interest, members of the CTFPHC should refrain from accepting more than $10,000 per year for testimony or review. |
| The Saudi Center for Evidence Based Health Care | RESEARCH SUPPORT：Non-monetary support valued at more than US $1000 overall (include equipment, facilities, research assistants, paid travel to meetings, etc.)；INVESTMENT INTERESTS Do you have current investments (valued at more than US $10 000 overall) in a commercial entity with an interest related to the subject of the meeting or work? Please also include indirect investments such as a trust or holding company. You may exclude mutual funds, pension funds or similar investments that are broadly diversified and on which you exercise no control |
| USPSTF | Any relevant financial disclosures valued at more than $1000 |
| WHO | The threshold for reporting relevant personal investment interests in the WHO declaration of interests form is 5000 United States dollars (US$): when the value of a single interest is below that amount, it does not have to be reported. Research support must also be disclosed, including non-monetary support valued at more than 1000 US$. |

*See Table 1 for the full names of the organizations

**eTable 6.** **Organizations’ policies on who should assess the disclosure of interests**

| **Organization*** | **Who should assess the DOI** |
| --- | --- |
| AAFP | Staff and the chair of the guideline development group |
| AAN | The following table provides details on who should e responsible for reviewing the submitted Relationship Disclosure Forms COI ** (Page 2 https://www.aan.com/siteassets/home-page/footer/about-the-aan/aan-relationships--conflicts-of-interest-policy.pdf) |
| AAO-HNSF | AAO-HNSF Ethics Committee |
| ACCF/AHA | Oversight committee |
| ACOG | The Chief Executive Officer, and shared with the volunteer leadership, ACOG staff, and other parties responsible for or involved in the relevant activity or subject, as needed |
| ACP | The DOI-COI Review and Management Panel |
| ACR | The Committee on Quality of Care chair |
| ADA | The Director, Center for Evidence-Based Dentistry and the Senior Vice President, Science and Professional Affairs in consultation with staff of the ADA Legal division |
| APA | The Conflicts of Interest Committee |
| ASCO | Chief Medical Officer or the Vice President and General Counsel, advised by the Associate Counsel for Ethics and Senior Director, Quality and Guidelines |
| ASPS | The ASPS President, the PSF President, the Executive Vice President and Chair of the Conflict of Interest Committee of the Society |
| ASTRO | The ASTRO Conflict of Interest Review Committee |
| ATS | Staff from the ATS COI management unit and ATS documents unit |
| AUA | Guidelines Oversight Committee (GOC), a three-member sub-committee from the PGC consisting of the Vice Chair of the PGC and two other members. |
| BASHH | The prospective development group |
| BHIVA | The chair and vice-chair of the BHIVA Guidelines Subcommittee |
| BTS | BTS Honorary Secretary and the Chair of the SOCC (BTS Standards of Care Committee) |
| CHEST | The Guidelines Oversight Committee (GOC) Chair and Vice-Chair, GOC Domain Liaison, and Panel Chair/submitter |
| CTFPHC | The Office of the CTFPHC reviews the disclosure forms in consultation with the chair or vice-chair of the CTFPHC to recommend the appropriate course of action |
| EAU | See details in Table 1. Assessment of COI prior to appointment of GO Board members, GO Panel (vice) Chairs and Panel members ** (Page 4 https://uroweb.org/wp-content/uploads/Managing-Conflict-of-Interest-06072017.pdf) |
| ENDO | The chair of the Clinical Guidelines Subcommittee |
| IDSA | The IDSA Executive Committee of the Board of Directors |
| JCQHR | Guideline Executive Committee |
| KNGF | The working group |
| NCEC | The reference person** (Page 6 https://assets.gov.ie/11496/4481f084d5a342ab8b10b8cc82bc2f10.pdf) |
| NICE | A senior member of the NICE guidance programme (or external contractor) |
| RCOG | Co-Chairs and if a second opinion is required, it is obtained from the Clinical Quality Board to whom the Guidelines Committee reports. |
| SCEBHC | The National Guideline Center oversight committee |
| SIGN | Written declarations of competing interests will be monitored by chairs of meetings of: • SIGN Council; • SIGN Executive Senior Management Team; • SIGN Subcommittees; • Strategy Group; • Guideline Programme Advisory Group; • Methodology Group; • GDG meetings |
| SIR | SIR staff and the Standards Division Councilor |
| USPSTF | Task Force Chairs |
| WHO | See Table 6.1. Declaration and management of conflicts of interest in guideline contributors** (Page 61 https://www.who.int/publications/i/item/9789241548960) |
| EHIF, University of Tartu, REMSA | The Guideline Advisory Board |
| *See the full names of the organizations in Table 1 of the main text  ** Details can be found at the web links | |

**eTable 7.** **Organizations’ policies on who should manage conflicts of interest**

| **Organization*** | **Who should manage the COI** |
| --- | --- |
| AAO-HNSF | The Academy Ethics Committee |
| AAOS | The chair of the external EBP committees |
| ACCF/AHA | Writing committee and Task Force Oversight Group |
| ACOG | The Chief Executive Officer |
| ACP | The DOI-COI Review and Management Panel |
| ADA | Evidence-Based Dentistry Center staff with the Panel Chair |
| APA | The APA Board of Trustees |
| ASCO | Questions about the application of this Policy Implementation will be decided by ASCO. ASCO will consider recommendations from the panel chair and co-chair and the Committee Chair (unless the question concerns their roles). |
| ASPS | Quality and Performance Measurement Committee, and ASPS COI Committee |
| ASTRO | ASTRO Conflict of Interest Review Committee |
| ATS | Guideline panel (co-)chairs, chairs will be advised by ATS staff in managing panel members with manageable conflicts of interest, and in summarizing management actions for ATS and within the methods section of the guideline. |
| AUA | Guidelines Oversight Committee |
| CCHMC | A development team (i.e., Stakeholders, Team Members, Patients/Family Members/Parents) |
| CHEST | the Chairs and GOC Domain Liaison |
| CTFPHC | The Office of the CTFPHC and the chair and vice-chair of the CTFPHC |
| EAU | COI Committee/Committee assigned by the EAU GO |
| ESGE | The ESGE Guideline Committee |
| JCQHR | The Guideline Executive Committee |
| NCEC | See Table 2: Frequency of making a declaration of interests and reference person ** (Page 6 https://assets.gov.ie/11496/4481f084d5a342ab8b10b8cc82bc2f10.pdf) |
| NICE | Senior member of NICE guidance programme or developer |
| SCEBHC | National Guideline Center |
| SIGN | SIGN Senior Management Team |
| SIR | SIR staff |
| USPSTF | Task Force Chairs |
| WHO | The responsible technical officer in consultation with the director of the technical unit |
| EHIF, University of Tartu, REMSA | The Guideline Advisory Board |
| *See the full names of the organizations in Table 1 of the main text  ** Details can be found at the web links | |

**eTable 8.** **Organizations’ policies on management of conflicts of interest**

| **Organization*** | **Basis for types of COI** | **How to manage COI** |
| --- | --- | --- |
| AAFP | NA | c. Review and Management of COIs Disclosures for each potential member will be reviewed by staff and the chair of the GDG prior to placement on the panel. If a disclosure is determined to represent a conflict of interest, potential actions include exclusion from the panel or limits on participation in discussions or voting on relevant recommendations. Each panel member will update any COI (verbally or in person) at each meeting of the GDG. d. Divestment Members of the GDG should divest themselves of financial investments they or their family members have, and not participate in marketing activities or advisory boards, of enti­ties whose interests could be affected by CPG recommendations.  e. Exclusions In some circumstances, a GDG may not be able to perform its work without members who have COIs, such as relevant clinical specialists who receive a substantial portion of their incomes from services pertinent to the CPG. |
| AAN | Severity of COI | See Identifying Relationships Considered Conflicting That Preclude Panel Involvement + Understanding Panel Composition and Responsibilities + Managing Conflicts: Restrictions for Conflicted Developers and Reviewers ** (Page 31-32 https://www.aan.com/siteassets/home-page/policy-and-guidelines/guidelines/about-guidelines/17guidelineprocman_pg.pdf) |
| AAO-HNSF | NA | The AAO-HNSF explicitly describes its COI policy for clinical practice guidelines in its Code for Interaction with Companies. Key points are (a) prohibiting direct company support for guideline development, printing, or publication; (b) disclosing all potential COIs of guideline development group members; (c) requiring that a majority of guideline development group members be free of financial COIs; (d) requiring that the chair must be free of financial COIs during guideline development and for 1 year after publication; and (e) disclosing all potential COIs of expert reviewers or advisers who are not officially part of the development group. Managing COI begins by distributing the completed contact and disclosure list to all guideline development group members for verification. All declared conflicts financial and intellectual are discussed with the group and published with the guideline. Serious or irreconcilable conflicts may result in dismissal of an individual from the development group. For each key action statement (recommendation), the group must decide if a member s COI requires him or her to be excluded from voting, recused from discussion related to that statement, or both. The COI disclosures should be reviewed and updated, as needed, during each conference call, at each in-person meeting, and prior to publication. |
| ACCF/AHA | NA | If a member of a writing committee has a relevant RWI regarding a product or competing product, and the section of the document relates to the specific or competing product, then the member is permitted to participate in the discussions but is not permitted to draft or vote on a recommendation or corresponding text.  If a member of a writing committee has a relevant RWI regarding a product or competing product, and the section of the document is not related to the specific or competing product, and the company does not manufacture or sponsor any relevant product/service or competing product/service, then the member is permitted to participate in the discussions and is permitted to draft and vote on a recommendation and/or corresponding text.  If a member of a writing committee has a relevant RWI regarding a product or competing product, and the section of the document relates to the company that manufactures or sponsors the product/service or competing product/service but not the specific product or class of products involved in their relationship, then the member is permitted to participate in the discussions but is not permitted to draft or vote on a recommendation and/or corresponding text. |
| ACOG | NA | Any Covered Individual who has an actual or potential conflict of interest in any proposed ACOG activity or subject shall remove him or herself from discussions and actions involving that subject, unless an ACOG vice president or senior staff person requests the Covered Individual to participate. |
| ACP | Severity of COI | See MANAGEMENT OF COIS ** (Page 358-359 https://www.acpjournals.org/doi/pdf/10.7326/M18-3279) |
| ACR | Severity of COI | The majority (at least 51 percent) of the guideline project development group must be free of conflicts of interest relevant to the subject matter of the project for at least one year prior to the LOI deadline and throughout the project until publication. Similarly, at least 51% of the Literature Review Team and at least 51% of the Voting Panel must have no conflicts of interest related to the subject of the guideline. NOTE: The 51% unconflicted threshold is a minimum; the ACR s goal will be to have significantly more than 51% unconflicted participants overall and on each smaller subgroup to allow for unanticipated midproject personnel shifts (e.g., a participant drops out due to illness or schedule changes). |
| ADA | Severity of COI | The following determinations of action will be made:  · No action. No disclosure or recusal necessary and individual may fully participate in the panel’s activities  · Information disclosure to expert panel. Individual must disclose potential conflict to the full panel and may fully participate in discussion and vote.  · Information disclosure to expert panel and recusal from voting. Individual must disclose potential conflict to the full panel and may fully participate in discussion but will be recused from voting.  · Recusal from all participation. Individual may not be part of the expert panel. |
| ASCO | Severity of COI and the personal role | Guideline Panels: a. Not Eligible to Serve on Panel; b. Eligible to Serve as Panel Chair or Co-Chair; c. Eligible to Serve in the Panel Majority. Clinical Practice Guideline Committee: c. Recusal  Guideline Advisory Groups: b. Eligible to Serve as Guideline Advisory Group Chair, Co-Chair, or Member ** (Page 3-7 https://www.asco.org/sites/new-www.asco.org/files/content-files/about-asco/documents/ASCO-Guidelines-COI-Policy-Implementation.pdf) |
| ASPS | NA | The Presiding Officer, with the assistance of legal counsel, may request the Volunteer Leader to: 1. remain in the room, participating fully in the discussion involving the conflict but not vote; 2. remain in the room while the matter is being discussed but not speak or vote; 3. provide his or her opinion and leave the room while the matter is considered and voted upon; 4. remain out of the room while the matter is being considered and voted upon. + ** (Page 5-6 https://www.plasticsurgery.org/Documents/Governance/conflict-of-interest-policy.pdf) |
| ASTRO | NA | At least 51 percent of those selected to serve on the panel will have no Financial Relationships with Affected Companies from the start of panel deliberations through publication of the Paper. For the remaining 49 percent, such Financial Relationships do not preclude panel membership, but, all such relationships must be carefully reviewed by the Panel Chairs to determine the extent to which the specific panelist is needed on the Paper, the impact of including/excluding the panelist and proposed methods to manage the potential COI. These determinations will be approved by the ASTRO Conflict of Interest Committee and ASTRO Legal Counsel. |
| ATS | Severity of COI | Based upon their disclosures, proposed panelists are considered as either free from relevant COI, having manageable COI that require management, or having disqualifying COI that must be terminated in order to serve as a member of the guideline panel. Experts in the subject matter of the guideline that have disqualifying COI may be permitted by the ATS to participate as a nonvoting expert contributor. ** (Page 2-5 https://www.thoracic.org/statements/document-development/resources/cpg-specific-coi-policy.pdf) |
| CHEST | Severity of COI and the personal role | See Appendix A: Categorization of Relationships and Activities by Role ** (Page 7-9 https://www.chestnet.org/-/media/chesnetorg/Guidelines-and-Resources/Documents/Guidelines-COI-Policy_Approved-January-2021.ashx) |
| CTFPHC | Severity of COI | See Table 2. Possible actions following disclosure of potential conflict of interest ** (Page 13 https://canadiantaskforce.ca/wp-content/uploads/2016/12/procedural-manual-en_2014_Archived.pdf) |
| EACTS | NA | Members with COI represent the minority of the Task Force and Chair(s) are free of COI. Additional measures used to manage COI include the multidisciplinary structure of Guideline Committees and the involvement of a clinical methodology expert or an independent EAO in the evidence synopsis and guidelines integration. |
| EAU | Personal role | See 6. Management and review of COI disclosure (high-risk COI) ** (Page 5-6 https://uroweb.org/wp-content/uploads/Managing-Conflict-of-Interest-06072017.pdf) |
| ENDO | Severity of COI | Managing Conflicts of Interest: The process of managing conflicts of interest is as follows ** (see Page 5-6 https://www.endocrine.org/-/media/endocrine/files/practice/cpg-coi-policy-2019-final.pdf) |
| ERA-EDTA | NA | ERBP aims that chairs and co-chairs are free of all conflict of interest and other guideline development group members have only minimal if any conflicts. If granted panel membership, individuals are not excluded from discussions or decisions, regardless of their potential conflict. Decisions are made in consensus and if no consensus can be reached, individual votes of the guideline development group members are reported to ensure complete transparency of process. |
| IDSA | Severity of COI and the personal role | Types of COI and requirements + Recusals ** (Page 16-18 https://idsociety.sharepoint.com/:b:/s/CAPG/EaXC004vbMdPna7PsrvQ34EBhl2yd5WOtyeUrEOK2rE2IQ?e=b9x6lT) |
| JCQHR | Scope of COI (Individual or Group) | 2) Management of and Response to COI ** (Page 11-12 http://minds4.jcqhc.or.jp/minds/guideline/pdf/MindsHB2014.pdf) |
| NCEC | Severity of COI | A range of actions/controls can be considered by the reference person (Table 2 refers) and they are: a. advice regarding confidentiality of documents under consideration b. on-going monitoring for any potential conflict c. denying access to relevant documents d. exclusion from specific discussions e. permission to participate in discussion when appropriate balance of opinion is available but exclusion from decision making ** (Page 6 Table 2: Frequency of making a declaration of interests and reference person https://assets.gov.ie/11496/4481f084d5a342ab8b10b8cc82bc2f10.pdf) |
| NICE | Severity of COI | If a member has an actual or potential conflict of interest the meeting chair should consider the following approaches and ensure that the reason for the chosen action is documented in minutes or records:  • requiring the member to not attend the meeting and potentially excluding the member from receiving meeting papers relating to their interest  • excluding the member from all or part of the relevant discussion and decision  • noting the nature and extent of the potential conflict of interest but judging it appropriate to allow the member to remain and participate. |
| SCEBHC | NA | Procedures for handling disputes in conflict of interest resolution: Final decision about inclusion and exclusion from a panel with rest with the NGC. Chairs of guideline panels will have to review individuals conflicts before each panel meeting and evaluate if the NGC should be involved based on new or changing conflict of interest. Once a member is approved for participation in a guideline panel, the panel chair will determine if specific panel members should be excused from individual recommendations or part of the discussion. |
| SIGN | NA | Where the level of declared interest could be seen to limit the independence of the individual, such individuals will not chair any SIGN meetings. Eligibility for GDG membership or peer review will be considered by the SIGN Senior Management Team on the basis of balance of risks and benefits and availability of alternative members or reviewers. |
| SIR | Severity of COI | B. Review and Categorization of Conflicts of Interest * (Page 2-3 https://www.sirweb.org/globalassets/aasociety-of-interventional-radiology-home-page/practice-resources/standards_pdfs/sir---cpg-coi-policy.pdf) |
| USPSTF | Severity of COI | Table 1. Description of Disclosures and Recommended Actions * (Page 6-7 https://www.uspreventiveservicestaskforce.org/uspstf/sites/default/files/inline-files/procedure-manual-2021_0.pdf) |
| WHO | Severity of COI and the personal role and the scope of COI (Individual or Group) | 6.9 Managing conflicts of interest at the individual level ** (Page 67-75 https://www.who.int/publications/i/item/9789241548960) |
| EHIF, University of Tartu, REMSA | NA | 1. Enforce the COI management strategy. 2. Stay alert to strong advocacy (e.g. aggressive argumentation, leading statements, repeating the same point). 3. When you detect strong advocacy, highlight it in front of the Panel and ask the person of interest to cease strong advocacy; also consider reminding the group about the specific COI in front of the Panel. 4. Repeat the statement about confidentiality (in particular, if the meeting is lasting more than one day). 5. Avoid statements that reflect your own views on the matters being discussed; aim to show neutrality. |
| NA: Not applicable  *See the full names of the organizations in Table 1 of the main text.  ** Details can be found at the web links. | | |
